# Data Sharing Models for the National Centralized Repository for Alzheimer's Disease and Related Dementias (NCRAD)

**DOI:** 10.1002/alz70855_106493

**Published:** 2025-12-24

**Authors:** Tatiana M. Foroud, Kelley M. Faber, Kaci Lacy, Corinne Kim, Kelly N. Nudelman, Jeffrey L. Dage, Kristen A. Russ, Michael C. Edler

**Affiliations:** ^1^ National Centralized Repository for Alzheimer's Disease and Related Dementias (NCRAD), Indianapolis, IN, USA; ^2^ Department of Medical and Molecular Genetics, Indiana University School of Medicine, Indianapolis, IN, USA; ^3^ Indiana Alzheimer's Disease Research Center, Indianapolis, IN, USA; ^4^ Stark Neurosciences Research Institute, Indianapolis, IN, USA; ^5^ Department of Neurology, Indiana University School of Medicine, Indianopolis, IN, USA

## Abstract

**Background:**

To advance research focused on Alzheimer's Disease and Related Dementias (ADRD) it is essential that a broad range of biological samples from well characterized research participants is widely available to the research community. To avoid costly repetition of experiments, and to support more advanced research utilizing information already gained from biological samples, it is critical that approaches be utilized to ensure that researchers share the data generated from valuable research samples.

**Method:**

The National Centralized Repository for Alzheimer's Disease and Related Dementias (NCRAD) is committed to ensuring that biological samples and the results from the study of NCRAD samples are made broadly available to the research community. NCRAD has developed detailed standard operating procedures and best practices that can be used by other biorepositories to support the broad sharing of data generated from biological samples.

**Result:**

NCRAD implements 3 key data sharing techniques with researchers receiving samples from the repository. The first is a material transfer agreement signed by all researchers receiving samples from NCRAD that commits them to share the data generated from their experiments with an approved data sharing resource. The second is the distribution of blinded samples, identified only with a unique barcode that cannot be linked to the clinical data for their research participant. A researcher is unblinded only after returning raw data to an approved data sharing resource. The third is a call held with the researcher before samples are distributed to discuss blinded data, determine the format of data return, and any other sample requirements. NCRAD has also developed web‐based catalogs that are used for studies that do not have a study website hosting clinical data and/or study results. To date, NCRAD has 13 catalogs available which can be used to select samples or to download data for subsequent analysis. All catalogs can be accessed only after the investigator signs a data agreement.

**Conclusion:**

NCRAD's commitment to making data generated from samples available to the broader community has played a pivotal role in advancing ADRD research in a number of different areas.

